# Assessing the potential long-term effects of sea-level rise on salt marsh’s coastal protective capacity under different climate pathway scenarios

**DOI:** 10.1007/s10661-024-12961-z

**Published:** 2024-08-16

**Authors:** Andre de Souza de Lima, Felicio Cassalho, Tyler W. Miesse, Martin Henke, Michelle R. Canick, Celso M. Ferreira

**Affiliations:** 1 Department of Civil, Environmental, and Infrastructure Engineering, George Mason University, 4400 University Dr, Fairfax, VA 22030 USA; 2 MD/DC Chapter, The Nature Conservancy, Bethesda, MD USA

**Keywords:** Chesapeake Bay, Maryland, SLAMM, Climate change, Hurricane Isabel

## Abstract

Salt marshes act as natural barriers that reduce wave energy during storm events and help protect coastal communities located in low-lying areas. This ecosystem can be an important asset for climate adaptation due to its particular capability of vertically accrete to adjust to long-term changes in water levels. Therefore, understanding marsh protection benefits thresholds in the face of sea-level rise (SLR) is important for planning future climate adaptation. In this context, the main goal of this manuscript is to examine how the storm protection benefits provided by salt marshes might evolve under SLR projections with different probability levels and emission pathways. In this study, a modeling framework that employs marsh migration predictions from the Sea Level Affecting Marshes Model (SLAMM) as parameterization into a hydrodynamic and wave model (ADCIRC + SWAN) was utilized to explicitly represent wave attenuation by vegetation under storm surge conditions. SLAMM predictions indicate that the SLR scenario, a combination of probability level and emission pathways, plays a substantial role in determining future marsh migration or marsh area loss. For example, results based on the 50% probability, stabilized emissions scenario show an increase of 45% in the marsh area on Maryland’s Lower Eastern Shore by 2100, whereas Dorchester County alone could experience a 75% reduction in total salt marsh areas by 2100 under the 1% probability, growing emissions scenario. ADCIRC + SWAN results using SLAMM land cover and elevation outputs indicate that distinct temporal thresholds emerge where marsh extent sharply decreases and wave heights increase, especially after 2050, and exacerbates further after 2080. These findings can be utilized for guiding environmental policies and to aid informed decisions and actions in response to SLR-driven environmental changes.

**Supplementary Information:**

The online version contains supplementary material available at 10.1007/s10661-024-12961-z.

## Introduction

Among the numerous threats climate change imposes on society, sea-level rise (SLR) stands out and tends to severely impact coastal areas (Grinsted et al., 2010; IPCC, 2021; Rajendra K Pachauri et al., 2014). Combined with this phenomenon, climate change can also exacerbate ongoing pressures on coastal communities, such as increasing the frequency and intensity of extreme events (Phillips & Crisp, 2010; Rangel-Buitrago & Anfuso, 2013), which can cause coastal flooding (Mcgranahan et al., 2007; Nicholls, 2004), induce sedimentary budget alterations (Esteves & Finkl, 1998), and result in underground water salinization (Su & Hock, 2016). Due to the projections for SLR, which can reach approximately 1 m in 2100 globally, the potential risks imposed on coastal communities and natural habitats are particularly significant in low-elevation areas (Hsiao et al., 2021; Li et al., 2020; Schuerch et al., 2018). Nonetheless, coastal ecosystems can attenuate the effects of climate change and, therefore, be an important asset for adaptation to these unprecedented challenges (Seddon et al., 2020).

Recent studies have pointed out the benefits of nature-based solutions (NbS) for coastal adaptation to climate change (Cohen-Shacham et al., 2016; le Coent et al., 2021). By definition, the concept has been employed as an umbrella term to highlight actions of management, protection, or restoration of ecosystems capable of providing environmental benefits and enhancing human well-being such as by attenuating waves, reducing erosion, contributing to long-term carbon storage, and adapting to SLR (Kabisch et al., 2016; McLeod et al., 2011; Sowińska-Świerkosz & García, 2021, 2022). For instance, coastal wetlands provide a series of ecosystem services and are responsible for USD 23.2 billion yr^−1^ in storm protection in the United States (Costanza et al., 2008). Moreover, coastal wetlands offer a series of additional ecosystem services over traditional gray engineering solutions (e.g., seawalls and breakwaters) such as providing nursery habitat and supporting recreational activities as well as maintaining water quality (Baptist et al., 2021; Bongarts Lebbe et al., 2021). Furthermore, a particular characteristic that distinguishes coastal wetlands, specifically salt marshes, from conventional engineering solutions is their adaptive capacity under different SLR scenarios, which is currently gaining momentum in the scientific community (Breda et al., 2021; Glick et al., 2013; Khojasteh et al., 2021; Leal Filho et al., 2022; Wu et al., 2017).

Salt marshes are coastal wetlands directly influenced by tides, usually located in low-elevation areas in sheltered estuaries, where the shoreline is protected from high-energy waves. Although threatened by the projected effects of climate change, this ecosystem can respond to SLR by inducing local surface elevation gains and migrating landward (Breda et al., 2021; Morris et al., 2002). This is accomplished as salt marsh vegetation has the ability to retain suspended sediments and organic materials available in the water column to vertically accrete. When this process occurs, the ecosystem can migrate landward and avoid its complete submersion (Fagherazzi et al., 2020; Raw et al., 2020). However, the horizontal migration might be limited by an anthropogenic physical barrier (i.e., “coastal squeeze”) or a sharp slope adjacent to the marsh area (Chávez et al., 2021; Torio & Chmura, 2013). Moreover, when the SLR rate surpasses the marsh’s rate of accretion, or there is insufficient sediment supply to enable a topographic compensation due to a change in sea level, an ecosystem conversion will be induced (Burns et al., 2021; Farron et al., 2020; Liu et al., 2021). Consequently, accelerated SLR may lead to a significant loss in coastal wetland areas around the globe (Bindoff et al., 2022). Understanding marsh resilience thresholds in the face of SLR is fundamental for correctly designing NbS for climate adaptation (Raposa et al., 2016; van Dolah et al., 2020).

Models that aim to simulate the ecosystem’s response to relative changes in sea level stand out as important tools to aid the decision-making process. Therefore, numerous studies have sought to respond to the increasing interest among stakeholders in understanding the impacts of climate change on salt marshes (Fagherazzi et al., 2020; Glick et al., 2013; Wiberg et al., 2020). Also, the salt marsh’s capacity to attenuate waves during storm surge events under both current and future scenarios has also been scientifically approached (Bigalbal et al., 2018; Gittman et al., 2014; Smith et al., 2018; Taylor-Burns et al., 2023). For example, Glass et al. (2018) and Paquier et al. (2017) evaluated the potential of marshes to attenuate storm surge water level during a 3-year monitoring campaign in the Chesapeake Bay and concluded that the storm surge reduction is greater in the marsh edge, due to changes in elevation. Besides that, Leonardi et al. (2018) also acknowledge that the salt marshes’ capacity to attenuate storm surges is dependent on marsh length due to bottom friction, usually implicitly represented in numerical models. It is known that the protection benefits provided by this ecosystem vary depending on different factors, but mostly water level conditions (Miesse et al., 2023; Zhang et al., 2020). Yet, analyzing the effectiveness of salt marshes as NbS under contrasting SLR projections is restricted in the literature, especially at a larger scale rather than exploring the behavior of individual wetlands. This lack of comprehensive understanding hinders the development of effective management actions and might lead to non-optimal restoration and adaptation practices (Thorslund et al., 2017).

In this context, the main goal of this manuscript is to examine how the storm protection benefits provided by salt marshes might evolve under various SLR projections with different probability levels and emission pathways. Here, we developed a modeling framework that employs marsh migration predictions from the Sea Level Affecting Marshes Model (SLAMM) as parameterization into a hydrodynamic and wave model (ADCIRC + SWAN) to explicitly represent wave attenuation by vegetation under storm surge conditions. To evaluate our framework, a study case for the Maryland counties of Dorchester, Wicomico, Somerset, and Worcester was developed by forcing ADCIRC + SWAN under current and future conditions. The potential effectiveness of salt marshes as NbS under contrasting SLR projections was assessed by comparing the estimated changes in wave heights during Hurricane Isabel.

## Methodology

### Current conditions in the study area

**Fig. 1:**
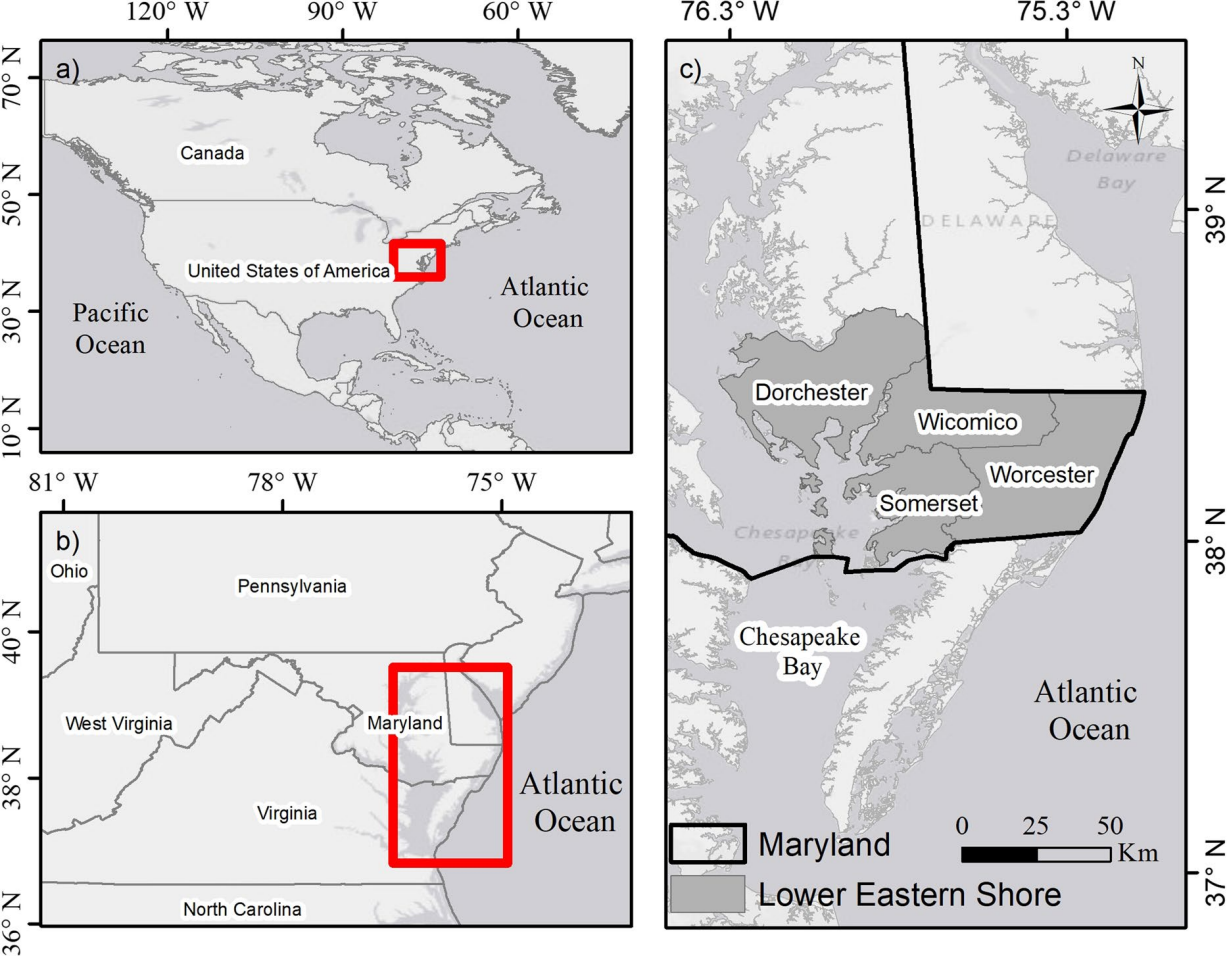
**a**–**c** Location of the study area in Maryland’s Lower Eastern Shore

The study area focuses on the Maryland counties of Dorchester, Wicomico, Somerset, and Worcester (Fig. 1), which, along with the surrounding greater Chesapeake Bay region, possess some of the highest rates of SLR on the Atlantic coast (Sallenger et al., 2012; Zervas, 2009) and the largest percentage of degraded salt marshes (Kearney et al., 2002). This region is also affected by widespread and considerable land subsidence (Karegar et al., 2016), which when combined with the already flat topography of the region can greatly amplify flood vulnerability (Eggleston & Pope, 2013; Johnston et al., 2021; Passeri et al., 2015). These combined environmental changes have already increased flood vulnerability to extreme storm events (Loftis et al., 2018) and inundation within low-lying areas under high tides (Eggleston & Pope, 2013). Consequently, this has resulted in many communities throughout the region now being identified at high risk of flood events (Spanger-Siegfried et al., 2014). Additionally, beyond human impacts, inundation and saltwater intrusion threaten the coastal ecosystem, causing habitat loss at rates untenable for marsh and wetland migration (Cassalho et al., 2023a, 2023b; Gedan et al., 2020). These trends are projected to continue into the coming decades, increasing the magnitude and frequency of flood events and placing the greater region at the forefront of coastal adaptation to climate change (Rezaie et al., 2021). Furthermore, despite the economic importance of the broad mid-Atlantic coast, these Maryland counties experience some of the lowest income rates within the state (U.S. Census Bureau, 2022) and are thus more vulnerable to flood impacts.

### Modeling framework

**Fig. 2:**
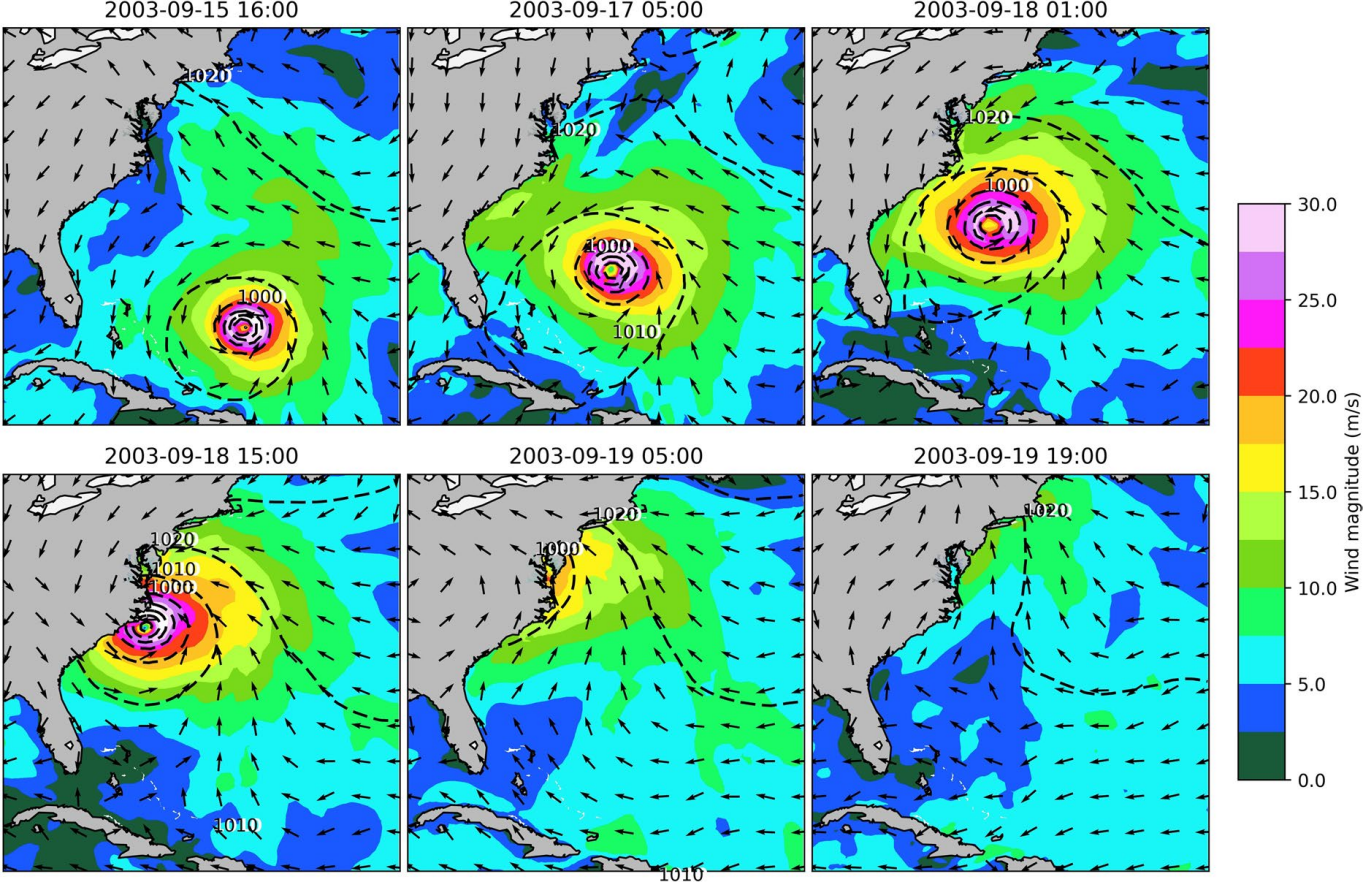
Progression of Hurricane Isabel under historical conditions, from 15 September 2003 to 19 September 2003

**Fig. 3:**
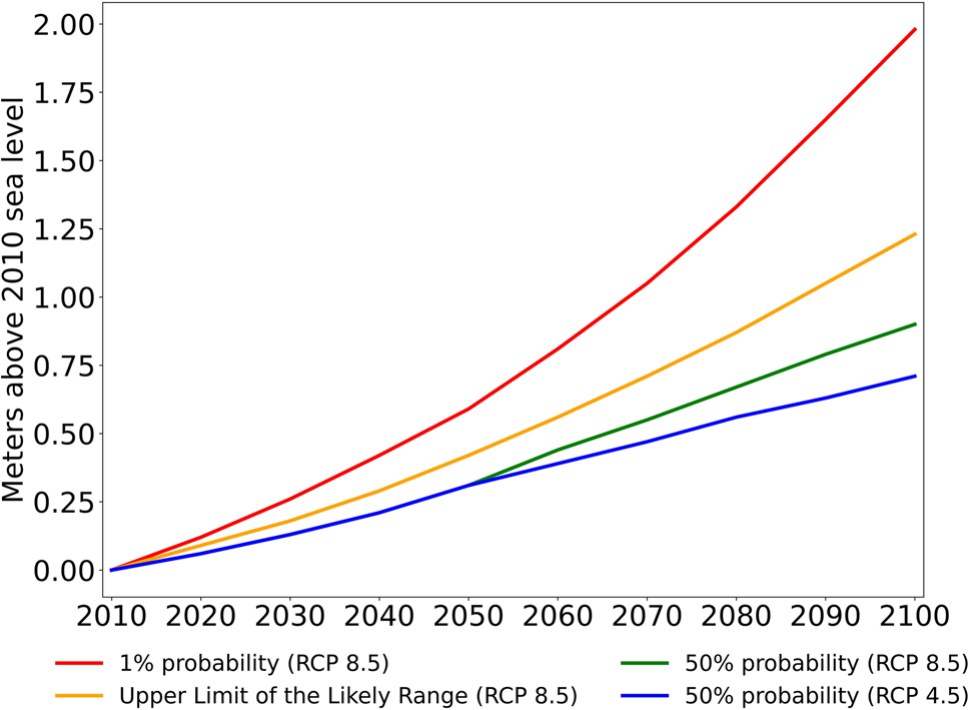
Sea-level rise scenarios based on a combination of probability levels and emission pathways

A modeling framework was devised employing results from a marsh migration model as parameterization into a hydrodynamic and wave model, capable of explicitly representing wave attenuation by vegetation. The marsh migration results identified future changes in land cover, including new salt marsh areas and marsh area losses in 2050, 2080, and 2100 due to four different SLR scenarios (Fig. 3). This framework was used to simulate a major storm event (Hurricane Isabel, Fig. 2), under current and future conditions, and the results were analyzed and summarized separately (“Marsh migration under different emission pathways” and “Effects of marsh migration on overland coastal waves” sections) and combined (“Changes in salt marsh area and wave impacts under different emission pathways” section) for each county in the study area (Fig. 1).

#### Marsh migration

The Sea Level Affecting Marshes Model (SLAMM) is a widely used model for projecting changes in coastal ecosystems under SLR conditions that has been applied in every US coastal state (Alizad et al., 2018). The simplest models of marsh migration, often referred to as “bathtub models,” predict marsh fate based on current marsh elevations compared to projections of future inundation with SLR. In addition to inundation, SLAMM addresses other nearshore processes important to marsh dynamics, including accretion, erosion, soil saturation, and (optionally) salinity (Clough et al., 2016). SLAMM incorporates these processes to predict future elevation relative to the local tidal range, which is used to determine the extent and frequency of saltwater inundation and therefore the type of wetland present.

Inundation in each model cell is calculated based on the cell’s minimum elevation and slope, where the corresponding rise of the salt boundary (the elevation that defines the boundary between salt marsh and dry land or fresh marsh) is tracked by reducing elevations of each cell as sea level rises, thus keeping mean tide levels constant at zero (Glick et al., 2013). Erosion is based on a maximum fetch threshold and proximity of the marsh to water; when the required wave conditions are reached, horizontal erosion occurs. Vertical accretion is given as a result of the buildup of organic and inorganic matter on the marsh’s surface. Accretion rates are specified for each marsh type and may be spatially variable based on salinity, distance to channel, or frequency of flooding/wetland elevation. Salinity is an optional mode within SLAMM that is particularly relevant for accounting for the effects of habitat switching in areas with significant freshwater flows. Lastly, saturation controls the migration of coastal swamps and fresh marshes onto adjacent uplands as a response of the water table to increases in mean sea level.

#### Hydrodynamics and waves

The Advanced CIRCulation (ADCIRC) model simulates water levels by solving the generalized wave continuity equation (GWCE) and currents by using the vertically integrated shallow water equation (Luettich et al., 1992). Simulating WAves Nearshore (SWAN) solves an Eulerian, phase-averaged, refraction model (Booij et al., 1999), providing wave propagation in irregular bathymetry and topography (Gorrell et al., 2011). Furthermore, using the coupled ADCIRC + SWAN model, the wave-current interaction, as described in Dietrich et al. (2011), provides a seamless exchange of hydrodynamic processes with the wave model, leading to enhanced precision in water levels, currents, and wave heights.

The numerical model ADCIRC + SWAN incorporates explicit vegetation characteristics in order to quantify how waves interact with vegetation. This approach uses the modified Dalrymple formula (Dalrymple et al., 1984) to estimate the average energy dissipation per unit area due to waves interacting with discretized vegetation, which is represented as rigid cylinders varying in densities, heights, and diameters (Mendez & Losada, 2004). This parameterization has demonstrated to possess superior accuracy in simulating wave attenuation compared to alternative methods (Baron-Hyppolite et al., 2019; Miesse et al., 2023). The calculation of wave attenuation by vegetation involves parameters such as bulk drag coefficient, vegetation density, height, and stem diameter, as well as water depth and wave height. This explicit representation within SWAN introduces a coefficient to the wave energy equation, influencing changes in wave frequency and direction based on the dissipation rate of total energy due to wave breaking and the total wave energy.

### Model setup

#### Marsh migration

The marsh migration predictions used in this study were generated by the application of SLAMM version 6.7 to coastal Maryland Clough (2021), using Maryland 2018 SLR Projections (Boesch et al., 2018), presented in Fig. 3. Each SLR scenario modeled consisted of a probability level and an emission pathway. All scenarios used the stabilized emission pathway (RCP4.5) through 2050. After 2050, the scenarios diverge to use either the stabilized emission pathway (RCP4.5) or the growing emission pathway (RCP8.5). Initial condition or “time zero (2010)” results were derived within SLAMM from land cover and elevation data subjected to tides only. Subsequent model time steps (2020–2100) applied SLR, erosion, and accretion. When an area fell below the modeled minimum elevation (relative to the tide) for its initial land cover class, it converted to another land cover class according to a flow chart of habitat transitions within SLAMM.

National Wetlands Inventory (NWI) data (U.S. Fish & Wildlife Service, 2020) was used as the wetland land cover input, with NWI wetland codes binned into SLAMM classes including three tidal salt marsh categories: regularly flooded marsh (RFM), irregularly flooded marsh (IFM), and transitional marsh. RFM includes low- to mid-elevation marshes that are inundated by tides at least once a day, while IFM includes high-elevation marshes that are inundated by tides once per day or less (Clough, 2021). Generally, RFM corresponds to a low marsh habitat dominated by Spartina alterniflora, and IRM corresponds to a high marsh habitat dominated by Spartina patens. However, since SLAMM classification does not incorporate specific vegetation data, field verification would be needed to confirm the habitat type of a particular area. Transitional marsh describes saltwater tidal scrub-shrub or forested wetlands, which in this study would include “ghost forests” that are in the process of transitioning to marsh. Other SLAMM land cover classes include non-tidal forested wetlands, tidal cypress swamps, inland fresh marsh, tidal forested wetlands, tidal fresh marsh, tidal flats, and open water. See Clough (2021), Appendix A, for a complete list of NWI codes that correspond to each SLAMM land cover class. Impervious land use was derived from Chesapeake Bay land use data (Chesapeake Bay Program Office, 2022) and classified as developed dry land in SLAMM. Forest cover was derived from Maryland tree canopy data (Dubayah et al., 2018) and used to split the typical SLAMM class of undeveloped dry land into two classes: forested dry land and non-forested dry land, which includes agriculture and turf.

Elevation inputs included the Coastal National Elevation Database–Topobathymetric Digital Elevation Model for the Chesapeake Bay (Danielson et al., 2016), and more recent county-specific LiDAR-based elevation data were available from the MD iMAP GIS data portal. Tide data were collected from NOAA tidal gauges with published datums and tide prediction tables. Elevation values were converted from NAVD88 to a tidal datum NOAA’s VDatum model (version 4.1.1), except for the area of Blackwater Wildlife Refuge, which was not covered by VDatum and where GPS observations were used instead (Hensel et al., 2008).

Erosion rates were derived from VIMS (2008) for most of the state, with Maryland Geological Survey (MGS, 2017) erosion rates used where available and (MGS, 2003) used for the coastal bays where it is the only available dataset. No erosion was assumed to take place in cells within 25 m of a shoreline erosion control structure. Based on the parabolic accretion-feedback curve, regularly flooded marsh (RFM) accretion rates range from 3 mm/year for marshes higher in the tidal frame to 6.5 mm/year for marshes lower in the tidal frame. The accretion rate was 3.2 mm/year in irregularly flooded marsh (IFM) and 3 mm/year in tidal fresh marshes. These values were defined based on local surface elevation table (SET) data from the Chesapeake Bay Sentinel Site Cooperative (CBSSC) Surface Elevation Inventory.

#### Hydrodynamics and waves

For the ADCIRC + SWAN model, a comprehensive numerical mesh was developed utilizing OceanMesh2D (Roberts et al., 2019) for the entire North Atlantic region, which is highly resolved for Maryland’s Chesapeake Bay area, where the resolution averages around 60 m overland. Elevation data sourced from county-specific LiDAR datasets (also employed in the SLAMM framework) combined with CoNED 2018 for the Chesapeake Bay region, whereas the US Coastal Relief Model (National Geophysical Data Center, 1999) and GEBCO 2020 (GEBCO Compilation Group, 2019) were utilized for the State and National scales, as well for the open ocean. Manning’s *n* roughness coefficients were attributed to non-salt marsh classes for friction value determination, as per Clough’s land cover data (2021), whereas field data campaigns provided quantitative data regarding vegetation densities, diameters, and heights for various marsh classes (Ferreira et al., 2022). A thorough overview of the modeling framework as well as a validation assessment for the numerical model outputs are outlined in Cassalho et al. (2022), where the coupled model was validated against seven water level stations and wave buoys within the Chesapeake Bay. An average RMSE of below 0.15 m was observed during a major hurricane event, and satisfactory wave results were obtained throughout the bay.

The aforementioned modeling framework was employed under current and future conditions in order to assess the wave-vegetation interactions in the study area, and Hurricane Isabel (Fig. 2) was selected as a representative case of a coastal extreme event. To numerically represent that event that occurred in September 2003, meteorological inputs, including horizontal wind velocity at 10 m above the surface and mean sea level pressure, were sourced from ECMWF ERA5 data (Hersbach et al., 2020). Moreover, tidal forcing was selected favoring eight major tidal constituents for the Chesapeake Bay due to its semi-diurnal tidal pattern (Khalid & Ferreira, 2020). Isabel became a Category 5 Hurricane on September 11, 2003, and made landfall a week later in North Carolina as a Category 2 Hurricane (Beven & Cobb, 2003). Among the most destructive storms to hit the East Coast in recent decades, Isabel claimed 16 lives and resulted in nearly $1.7 billion in insured property damage, with Maryland bearing almost a quarter of that total (Post & Schuh, 2005). In order to thoroughly represent distinct future settings, the initial water surface above the geoid was adjusted in ADCIRC + SWAN, and Hurricane Isabel was also simulated under four distinct SLR scenarios based on the Representative Concentration Pathways (RCP) 4.5 and 8.5, varying from the year 2010 to the year 2100 (Boesch et al., 2018). Moreover, the model was also parameterized based on the SLAMM outputs. In Fig. 3, the blue curve is based on RCP 4.5 and represents a 50% probability that SLR meets or exceeds the estimated value for the SLR projection, while the green line represents the same probability for RCP 8.5. On the other hand, the orange and red curves represent a 17% chance that SLR will meet or exceed the projected value (i.e., the upper limit of the likely range) and the worst-case scenario modeled (1% probability), respectively, both based on RCP 8.5 (Fig. 3).

## Results

### Marsh migration under different emission pathways

**Fig. 4:**
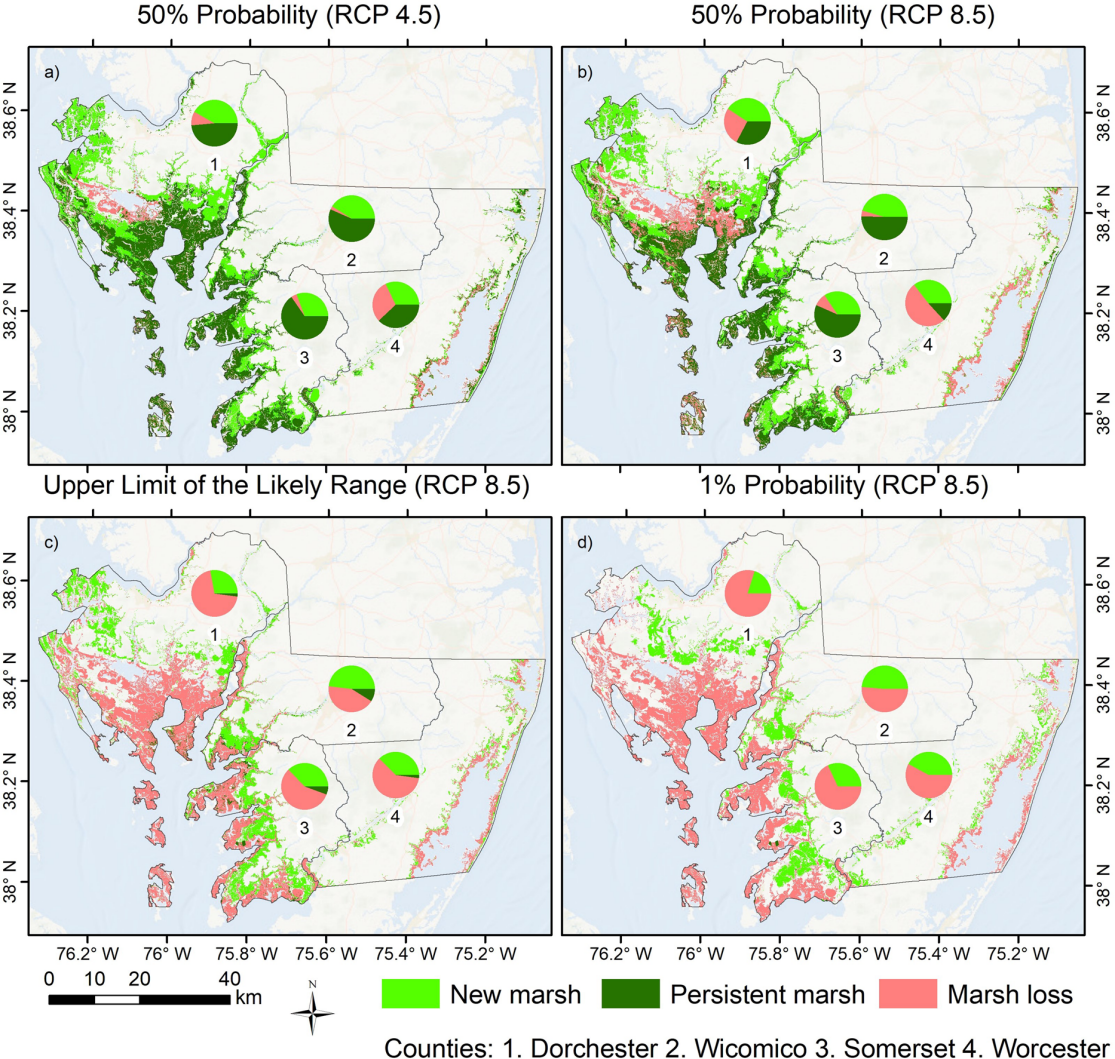
SLR-driven changes in marsh areas based on multiple emission pathways by 2100

SLAMM predictions indicate that the SLR scenario, including the probability level and emission pathways, plays a substantial role in determining future marsh migration or area loss. For example, results based on the 50% probability, stabilized emissions scenario (RCP 4.5) (Fig. 4a) show an increase of 45% in the marsh area on the Lower Eastern Shore by 2100 (Supplementary Material 2). In Dorchester County, where half of the marshes in the region are currently situated, the changes due to marsh migration over time stand out. For the same county under the same stabilized SLR scenario, our analysis reveals a net increase of 186.1 km^2^ in the total area from 2010 to 2100, with an original area of 336.8 km^2^ expanding to 522.9 km^2^ by the end of the century. This change is attributed to a substantial gain of 241.9 km^2^ by 2100 derived from multiple classes being expected to convert into salt marshes, including forested dry land (78.5 km^2^), non-forested dry land (71.7 km^2^), non-tidal forested wetland (46.6 km^2^), and tidal forested wetland (45.1 km^2^). On the other hand, a loss of 55.8 km^2^ from the original marsh area is also predicted, of which 91% is projected to be converted into open water and 9% into unvegetated mudflats in the same county. Regarding the persistent marshes, three different salt marsh classes from SLAMM are generalized in Fig. 4 (i.e., regularly flooded marsh, RFM; irregularly flooded marsh, IFM; and transitional marsh, TM) in order to facilitate result visualization. However, it is important to mention that although approximately 50% of the existing marsh area from 2010 is projected to adapt to SLR by 2100 (i.e., persist or migrate), significant ecological changes might occur due to the fundamental variations in the existing hydrological regimes due to rising sea levels. While less than 10% of the total marsh area was classified as RFM in time zero, approximately half of the total area is categorized under the same class by 2100, mostly converted from IFM.

The growing emission pathway (RCP 8.5) was analyzed based on three distinct probability levels: central estimate or 50% probability (Fig. 4b), the upper limit of the likely range or 17% probability (Fig. 4c), and the 1% probability (Fig. 4d). Considering the 50% probability of the growing emissions scenario (Fig. 4b), the Lower Eastern Shore still shows a 27% increase in total marsh area by 2100, despite a SLR increment of 0.19 m in contrast to the stabilized emissions scenario (see blue and green lines in Fig. 3). Although changes in marsh area for Somerset and Wicomico counties are relatively minor and Dorchester County shows a total loss of nearly 60% in contrast with the stabilized emissions SLR scenario, the overall result for the three aforementioned counties still reflects a net gain in terms of marsh area. Conversely, in Worcester County, the water level increase due to SLR led to a total marsh loss of approximately 59 km^2^, thus, accounting for a decrease of approximately 25% compared to 2010, making it the only county to experience a net loss in its marshland area by 2100 for this scenario (Supplementary Material 3). Notably, from this loss, approximately 90% is undergoing conversion to open water, while the remaining 10% is being altered into unvegetated mudflats. Moreover, the contribution of new marsh areas, totaling approximately 40.7 km^2^ (73% of total marsh area by 2100), includes land transformations from forested dry land (15.4 km^2^), non-forested dry land (15.1 km^2^), tidal forested wetland (5.9 km^2^), and non-tidal forested wetland (4.3 km^2^). Lastly, only a quarter of its original area is projected to persist until the end of the century, although being almost completely converted to RFM (78% of the county area), whereas IFM and TM represent 18% and 4%, respectively.

SLAMM results based on the upper limit of the likely range, growing emission scenario (Fig. 4c), show a very distinct outcome in comparison to the previously described results. In this case, SLR reaches 1.23 m by 2100, resulting in a net loss of nearly 45% of the marsh area in Maryland’s Lower Eastern Shore. Furthermore, the region is projected to lose almost 15% of its dry land areas, in addition to the already mentioned marsh reduction (Supplementary Material 4). Although projected to lose a substantial portion of its marshland by 2100, accounting for around 42 km^2^, Wicomico stands out in this scenario as the only county with a net gain in the marsh area, due to a conversion of approximately 47 km^2^ of forested dry land (40%), non-forested dry land (29%), non-tidal forested wetland (28%), and tidal forested wetland (2%) into salt marshes. In this instance, 58% of new marsh areas are classified as RFM, whereas 24% are classified as IFM and 18% as TM. On the other hand, the full extent of salt marshes projected to adapt to SLR in Wicomico County are converted to RFM.

Finally, the 1% probability, growing emission scenario (Fig. 4d) depicts the ecological effects of an extreme SLR projection in the region, where SLAMM results predict a total reduction of about one-third of the region’s overland territory (Supplementary Material 5). In this instance, all counties are projected to experience a net loss in marsh areas, and only 1% of the existing marshes are expected to adapt to future conditions and compose the remaining marshland in 2100, accounting for 556 km^2^ less than in the first scenario. While SLR may lead to new marsh areas through habitat conversion even in the most extreme circumstances, it is important to note that this is expected to be less substantial than in other scenarios. The extent of habitat conversion into salt marshes due to SLR is projected to be 30% smaller than in the most optimistic scenario and about 18% smaller than the upper limit of the likely range scenario. Moreover, in this case, new marshes are expected to be converted from the same classes as in the other scenarios, albeit in different proportions: non-forested dry land (116 km^2^), forested dry land (85 km^2^), non-tidal forested wetland (75 km^2^), and tidal forested wetland (2 km^2^). In this 1% chance of occurrence scenario, Dorchester County stands out for having approximately half of its territory converted into flooded areas. Also, the county is projected to experience a 75% reduction in total salt marsh areas by 2100 when compared to current conditions, even though a substantial increase of 30% is expected by 2050.

### Effects of marsh migration on overland coastal waves

**Fig. 5:**
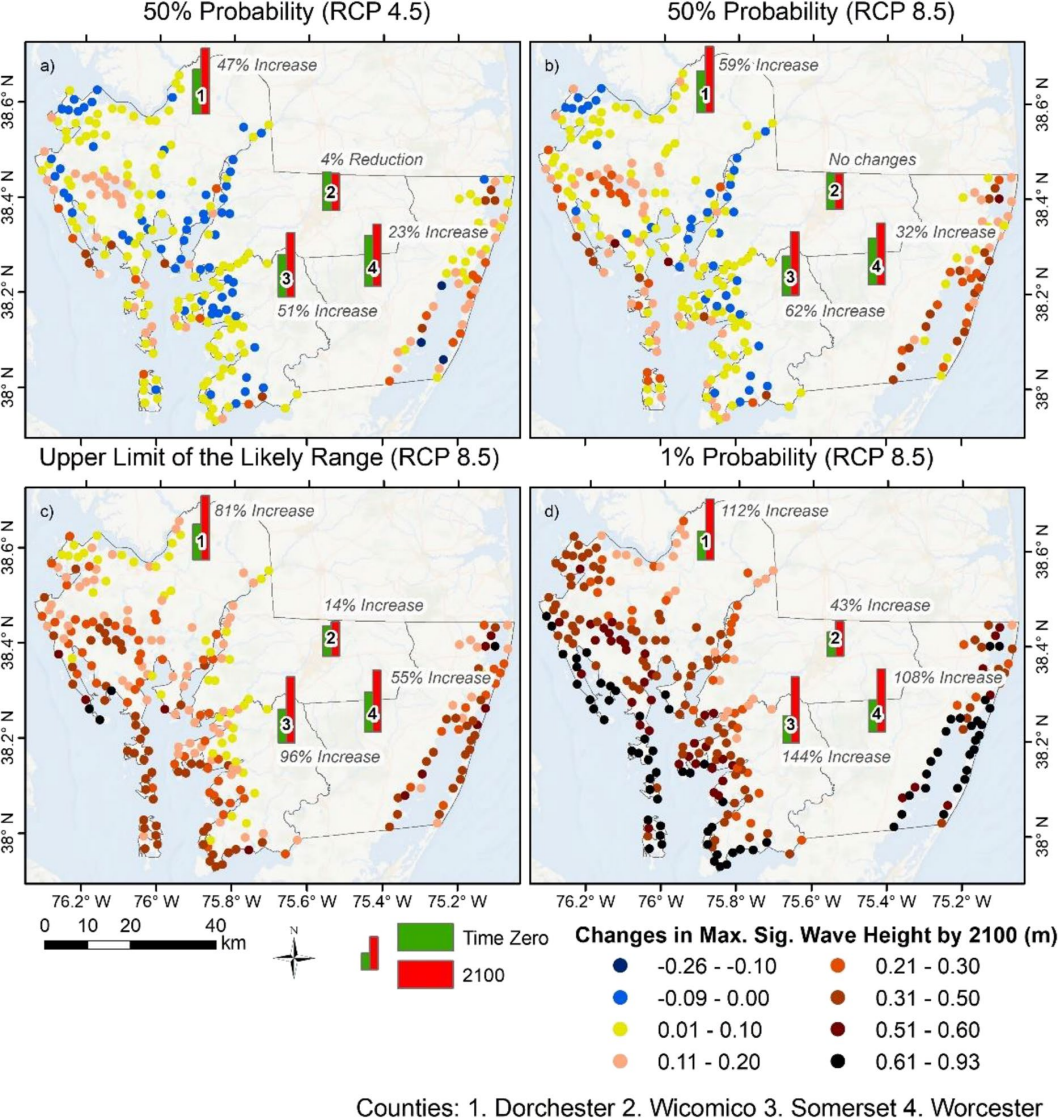
Changes in Hs during Hurricane Isabel when simulated under different SLR scenarios by 2100

In order to simulate the effects of Hurricane Isabel under current and future conditions and investigate the dependency of future coastal protection to changes in land cover, the previously discussed SLAMM predictions were combined with their respective increments in water levels (i.e., SLR scenarios) as model parameterization, from the selected emission pathways (Fig. 3). In this study, only waves that reached overland areas (i.e., areas that are normally afloat under regular meteorological conditions as of the initial marsh extent) were accounted for. Factoring that in, Hurricane Isabel produced considerable waves over the entire Maryland’s Lower Eastern Shore. Notably, during that extreme event, waves up to 0.65 m reached overland areas in the coastal bays of Worcester County and 0.5 m in Wicomico County, although waves of 0.2 m were at the 90th percentile in Worcester, twice as high as in the waves reaching Wicomico County during the same event (Supplementary Material 2). Regarding the Somerset and Dorchester counties, the average values for maximum significant wave height (Hs) were both below 0.10 m, with maximum values of 0.55 and 0.58 m, respectively. Figure 5 shows potential future changes in Hs based on different SLR scenarios and their effects on marsh migration. The dots on the map represent the numerical mesh nodes that define the coastline during the baseline scenario (current conditions), where the changes in Hs were calculated in contrast to the future scenarios. Furthermore, the overall changes in maximum Hs per county are also represented by single bar plots.

Figure 5a shows the impacts of Hurricane Isabel under the influence of the stabilized emission pathway employed in this study, tied to a 0.71 m increase in water levels due to SLR. In this case, the individual maximum Hs values reach up to 0.85 m in Dorchester County, representing a 47% increase compared to the baseline scenario. Worcester stands out with the most substantial changes in Hs by 2100. Despite the fact that the maximum values are similar for Dorchester, Somerset, and Worcester counties, the latter holds 0.4 m waves at the 90th percentile. On the other hand, Wicomico County shows a 4% reduction in Hs, where those changes can be visualized along its entire coastline. The second scenario presented in Fig. 5b shows the influence of the likely range of a growing emission pathway, in which a 0.9 m increment in MSL is projected by 2100 due to SLR. When Hurricane Isabel is simulated under these conditions, the differences in the maximum individual values are moderate, and the increases in each county vary up to 12% higher than in the previous scenario, as in Dorchester County, where maximum Hs values in overland areas could reach 0.92 m (i.e., 8% higher), although 0.21 m waves represent the 90th percentile in the county (i.e., 31% higher). The most substantial changes can be visualized in the Coastal Bays, in Worcester County. In that instance, maximum Hs values of 0.86 m could reach overland areas and an increase of 35% in the values that fall within the 90th percentile compared to the previous scenario. No changes can be visualized between waves generated during Hurricane Isabel under current and future conditions for this specific case in Wicomico County.

The same emission pathway (RCP 8.5) was employed in another two different probability levels that, if combined with Hurricane Isabel, could potentially generate substantial impacts in most of the study area. Figure 5c shows the projected waves based on a 1.23 m increase in water levels due to SLR. In this instance, Dorchester, Somerset, and Worcester counties show maximum Hs values above 1 m. Somerset County stands out with an almost 100% increase in maximum Hs, where areas that would be afloat under normal current conditions could face waves up to 1.08 m by 2100 during such an extreme event, approximately a half-meter increase (Supplementary Material 4). However, in terms of overall changes, Worcester County shows the maximum value within the 90th percentile among the cases, with a 0.66 m Hs. Regardless of a considerable increment in water levels during an extreme event, Wicomico County shows a modest increase in wave heights, reaching mean values near 0.15 m (i.e., two times higher than the wave impacts under current conditions). Finally, the 1% chance of occurrence shows the projected effects of Hurricane Isabel under a substantial increment of 1.98 m in water levels due to SLR. For this particular scenario, all four counties show an increase of at least half a meter in the individual maximum Hs values (Fig. 5d), as well as average values reaching up to 0.48 m in Worcester County, although 0.93 m waves fall within the 90th percentile. Under these extreme conditions, even Wicomico County could reach waves up to 0.7 m, more than three times higher on average (Supplementary Material 5).

### Changes in salt marsh area and wave impacts under different emission pathways

**Fig. 6:**
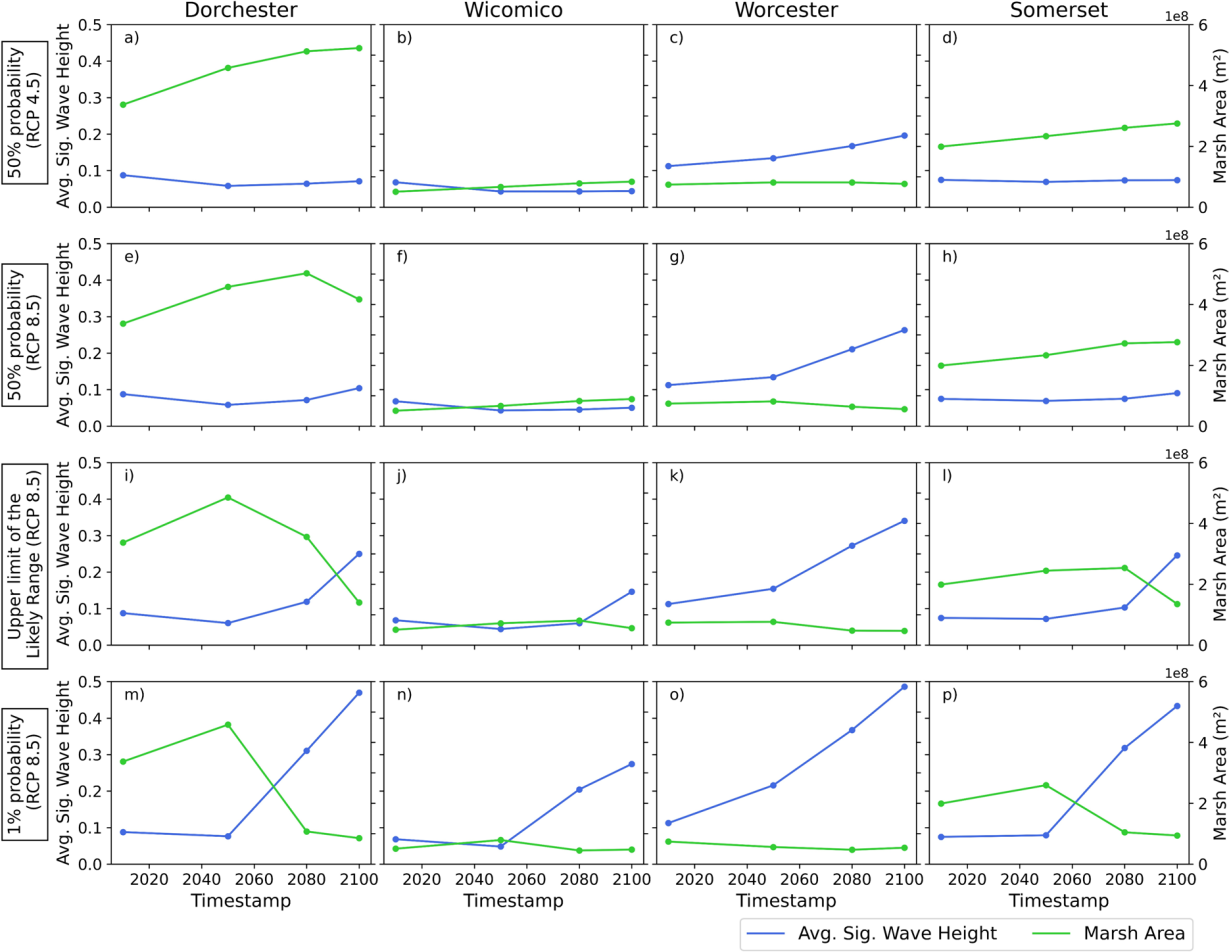
Comparison between salt marsh area changes due to SLR-induced migration and potential shifts in average of the maximum Hs during various Hurricane Isabel simulations under future scenarios

Figure 6 conveys a summary of changes in the total salt marsh area (i.e., RFM, IRM, and TM combined) due to SLR-induced habitat transitions in contrast with potential changes in the average of maximum individual Hs values throughout the study area, when Hurricane Isabel is simulated under multiple future scenarios. Each subplot informs those changes in four different timestamps (i.e., time zero, 2050, 2080, and 2100) for each county in Maryland’s Lower Eastern Shore. Regardless of the SLR scenario, a consistent inverse relationship can be visualized throughout the time series, where decreases in salt marsh areas are nearly proportional to increase in wave heights. Under the 50% probability, stabilized emissions scenario, this becomes particularly apparent in Dorchester County (Fig. 6a) where a 36% increase in marsh area by 2050 corresponds to a 34% reduction in the average of the maximum wave heights throughout the county. The same county shows a 55% increase in marsh area by 2100 and an overall 20% reduction in the average of the maximum wave heights by the end of the century. Although less substantial in terms of change magnitudes, Wicomico County also follows the same overall pattern (Fig. 6b). In this instance, a crossover point can be visualized by 2050, where a 31% increase in total salt marsh area corresponds to a 37% reduction in wave heights. The average of the maximum Hs remains lower than in the baseline scenario, and there is no change in the 90th percentile when the same scenario is compared with the results for the 2100 projection. Similarly, changes in total salt marsh area reach a 17% increase by 2050 in Somerset County, and a positive trend in this variable is evident in the entire time series. Thus, in this scenario, negligible changes in the average of the maximum Hs are projected by the end of the century. On the other hand, Worcester is the only county in the study area to not follow the same pattern, where a slight reduction in marsh area is projected, and the average of the maximum Hs substantially increases after 2050 (Fig. 6c).

The same pattern is also evident in the 50% probability, growing emissions scenario, although changes in both variables diverge and become more accentuated after 2080. In Dorchester County, for example, a 17% decrease in marsh area is projected after 2080 in this particular scenario, thus corresponding to a 45% increase in the average of the maximum Hs for the same period (Fig. 6e). In Somerset County, where a positive trend in total salt marsh area was evident in the previous scenario, a slight disruption is also apparent after 2080 (Fig. 6h). In this instance, although the salt marsh area stabilizes, a 20% increase in average of the maximum Hs is projected. While negligible changes are anticipated for Wicomico County when compared to the stabilized emission pathway scenario, Worcester County starts showing a negative trend in the salt marsh area after 2050, when the values for average of the maximum Hs reach 0.21 m by 2080, twice as much as in the baseline scenario (Fig. 6g).

When the growing emission pathway is analyzed under a different probability level (i.e., the upper limit of the likely range), a considerable difference in magnitudes and patterns is observed, also more prominent after 2080. That is, as salt marsh area is reduced, increases in wave heights are noticeable. In Somerset County, where changes in the salt marsh area would stabilize in the previous scenario towards the end of the century, a nearly 50% reduction is projected after 2080, and in this case, a crossover point is noticeable, when the average of the maximum Hs is 138% higher than in the previous timestamp (Fig. 6l). In Dorchester County (Fig. 6i), a reduction in total salt marsh area is now projected to occur after 2050 and become more substantial after 2080, where a crossover point is also noticeable. By the end of the century, salt marshes could face a nearly 60% reduction in total area compared to current conditions, with waves reaching 0.25 m over the sampled area during such a storm event. Differences in both variables are also considerable even in Wicomico County, where a 30% reduction in marsh area is projected after 2080 and a respective 140% increase in the average of the maximum Hs is expected (Fig. 6j). In Worcester County, the pattern remains the same, although changes in magnitude are noticeable after 2050 (Fig. 6k).

The effects of SLR on both variables are considerably exacerbated under the 1% chance of occurrence scenario, and the potential impacts that were more prominent after 2080 have now shifted and are expected to start happening 30 years earlier. A 76% reduction in salt marsh area is projected in Dorchester County after 2050, and by the end of the century, the county could lose three quarters of this ecosystem (Fig. 6m), and waves could reach nearly half a meter on today’s overland areas. However, even under such extreme SLR rates, salt marshes could prosper and grow by 2050 in Dorchester, Wicomico, and Somerset counties (Fig. 6m, n, and p, respectively), despite being drastically suppressed in the years after. In the same counties, the most substantial increase in the average of the maximum Hs happens by 2080 when modeled mean values are close to three times higher than in the previous timestamp, reaching up to 0.47 m in Dorchester County in 2100 (Fig. 6m). In Worcester County, a reduction in salt marsh area is apparent in the entire time series, and by the end of the century, the county could lose approximately 30% of this ecosystem under this specific emission pathway scenario. The average of the maximum wave values exponentially increases throughout the entire analyzed period, and the highest average value for the study area is projected (Fig. 6o).

## Discussion

### Changes in salt marsh area and habitat trade-off

The existing approaches to estimate the dynamics and conversion of coastal wetlands into different ecosystems vary in scale, complexity, and the number of uncertainties that are accounted for (Fagherazzi et al., 2012; Mcleod et al., 2010). However, they should incorporate the main processes in coastal wetland evolution such as erosion and accretion rates, inundation, salinity, and soil saturation (Clough et al., 2016; Nymanl et al., 1993; Turner et al., 2004) to avoid overestimating marsh vulnerability to SLR (Kirwan et al., 2016a, 2016b). In this context, numerous attempts to model marsh migration and coastal habitat trade-offs stand out in the literature. For example, Rogers et al. (2012) utilized empirical data to develop a landscape-scale model to estimate the response of coastal wetlands to changes in water level and compared their framework estimations to a bathtub model. Their results highlight the importance of utilizing wetland relative elevation for a more accurate prediction and to better inform decision-makers, given that a simplistic bathtub approach will overestimate the loss of wetland extent due to SLR. Additionally, Kirwan et al., (2016a, 2016b) proposed a modeling framework to account for seaward edge erosion and vertical soil accretion to explore marsh response to changes in water levels. In this study, it is emphasized that salt marshes can migrate to new areas and adapt to SLR even under accelerated rates, regardless of area losses near the salt marsh edge. However, Ge et al. (2016) emphasize that sediment supply is key for coastal resilience, and ideal conditions for marsh migration can also be tested and modeled. The authors developed a local scale model to assess the role of sediment accretion and how that affects marsh adaptation to a range of potential future conditions.

The SLAMM projections employed in this study reveal that regardless of the SLR rates, new salt marsh areas are projected to be created at the expense of four different environments: forested dry land, non-forested dry land, non-tidal forested wetland, and tidal forested wetland (Supplementary Material 6). In this setting, non-forested dry land could have up to nearly 10% of its total area (147 km^2^) converted into salt marshes by 2100 under the 50% probability of the RCP 8.5 scenario (Fig. 4b) and 8% of its total area (116 km^2^) under the most severe scenario. Moreover, tidal forested wetlands could have up to 72% (78 km^2^) of its original area converted into salt marshes under the 50% probability, stabilized emissions scenario (Fig. 4a). Although this class is projected to be severely impacted by SLR under the 1% chance scenario, where nearly 95% of its total area would be submerged by water, approximately 2 km^2^ would be converted into salt marshes.

It is worthwhile to point out that this present analysis assumes a best-case scenario for salt marshes, being that all agricultural, forested, and non-developed dry land within the specified tidal range could successfully be converted into wetlands since no potential future barriers were included in the model to simulate a “coastal squeeze” effect. In a similar approach to investigate the future salt marsh’s response to SLR, Raw et al. (2020) predicted a potential 40% loss in marsh area in South Africa due to limited habitat for marsh migration. To address a potential underestimation of these values, the authors propose identifying areas within the tidal frame that are designated for development plans. This approach allows environmental managers to proactively flag potential marsh loss resulting from future urbanization. Moreover, marsh area loss is imminent when ecosystem connectivity is interrupted either by natural or human barriers. Thus, indicating the importance of mapping and preserving areas where marsh migration can occur to inform managers and ensure migration corridors will not be disrupted to the detriment of coastal infrastructure that reduces short-term coastline retreat (Vinent et al., 2019).

The results also indicate that in the study area, a potential resilience threshold can be more closely associated with the SLR probability level and emission pathway than with a specific timestamp. For example, under both 50% probability scenarios (Fig. 4a, b), a net gain in terms of total salt marsh area is expected by the end of the century in Dorchester, Somerset, and Wicomico counties, while only a 24% reduction in marsh areas in Worcester. On the other hand, total marsh areas are projected to significantly decrease under the more severe scenarios (Fig. 4c, d), and in those cases that could start happening after 2050. Under those extreme probability levels, it is noteworthy that despite the high SLR rates, a substantial increase in salt marsh areas is evident by 2050. For instance, in Dorchester County, a maximum peak in terms of area is reached under the first SLR scenario (Fig. 4a) by the end of the century (522 km^2^), whereas a peak of similar magnitude is also reached under the 1% probability scenario, although 50 years prior, thus, indicating the relevance of utilizing a comprehensive range of SLR scenarios in marsh migration modeling.

Comparatively, a similar study in the Chesapeake Bay projects total salt marsh areas to be nearly four times larger than historical observations by 2100 and indicates that forested wetlands would be largely converted into salt marsh, even when accretional and erosional processes were completely neglected (Molino et al., 2022). Conversely, Nunez et al. (2021) examined two representative locations in the Chesapeake Bay with distinct physical settings (i.e., a developed, high topography and a natural, low topography with steep banks). Their marsh migration results under moderate and severe climate scenarios indicate that, despite the SLR rates, marsh reduction can be substantially dependent on upland bank conditions. In their study site, which is developed and has high topography, salt marshes can be suppressed by nearly 90% under extreme SLR scenarios. Their study also indicates that the emergence of new salt marsh areas primarily occurs where forests and shrublands start to experience inundation. These disparities in numbers, even when not substantial, indicate a need to also investigate the uncertainties in marsh migration modeling. The SLAMM model results, for example, in general show reasonable agreement with different deterministic models and consistent results when compared to observations over decadal timescales, gradually decreasing over time (Mogensen & Rogers, 2018).

### The role of salt marshes in protecting coastal areas

It is evident that distinct temporal thresholds emerge where marsh extent sharply decreases and wave heights increase. Yet, regardless of the underlying factors that drive these consistent patterns that repeat under different SLR scenarios, these critical points might indicate essential resilience thresholds, thus, being useful for environment and coastal managers, decision-makers, and county authorities, informing their strategies and actions in response to climate-driven environmental changes. The relationship between salt marsh extent and wave height emerging from the results section reveals a connection between those two variables, although examining the reasons for this connection falls outside the scope of this study.

In the literature, two main approaches have emerged to investigating and quantifying wave attenuation by vegetation, and how salt marshes can contribute to coastal protection under storm surge conditions. The first, which is more prevalent and established, delves into the interaction between waves and salt marsh vegetation at a local scale, employing different techniques such as field-based empirical and numerical model investigation (Garzon et al., 2019a, 2019b; Garzon et al., 2019a, 2019b) and distinct numerical techniques to incorporate vegetation into modeling frameworks (Baron-Hyppolite et al., 2019; Miesse et al., 2023). Those studies also aim to understand how different salt marsh species behave under distinct water levels and wave conditions during storm surge events (Cassalho et al., 2023a; Coleman et al., 2023) and to quantify erosion at the salt marsh edge due to wave impacts (Bendoni et al., 2019; Tonelli et al., 2010). These studies have convincingly demonstrated the significant role played by salt marsh vegetation in attenuating waves, although lacking a comprehensive view in terms of identifying regional patterns on how this type of vegetation can protect coastal communities during extreme events and how this can evolve in the coming decades. SLAMM predictions have also been employed as model parameterization to the XBeach model, in order to assess changes in wave attenuation under future scenarios (Hijuelos et al., 2019). The study highlights how vegetation type affects wave attenuation in changing wetland landscapes and how coastal protection can critically change with reduction in wetland areas. Similarly, Castagno et al. (2022) evaluated wave reduction as a function of marsh restoration to highlight how a vegetated marsh can substantially reduce wave energy, even under future conditions. More recently, researchers have begun to adopt a more generalized approach (Cassalho et al., 2023b; Miesse et al., 2023), but this poses limitations in terms of parameterizing vegetation characteristics and the accuracy of results, particularly in regional studies, which are still limited in the literature.

For that reason, the scope of this study focuses on identifying spatiotemporal patterns on how marsh extent and wave impact might evolve under different SLR scenarios, rather than quantifying wave attenuation by vegetation, a subject already explored by other studies on both local and regional scales. Thus, empowering decision-makers to take timely action before this invaluable ecosystem collapses or ceases to provide these invaluable protection services. Therefore, it is clear from Fig. 6 that even for the mildest SLR scenario, wave heights start to increase significantly after 2050 and intensify after 2080. These consistent patterns seem to be true for most counties in all scenarios, especially in Dorchester and Somerset counties, where salt marshes are more abundant. As previously mentioned, the marsh extent curve appears to be more challenging to predict, as the changes in marsh area, although driven by the same process (i.e., SLR), depend on the availability of land that can be claimed by the marsh, which is county-dependent. However, it can be observed that 2050 seems also to be a reasonable threshold for salt marsh extent collapse for the most drastic SLR scenarios. Therefore, indicating that under those conditions, decision-makers must act in a timely matter, as marsh loss is often irreversible (Matthew L. Kirwan et al., 2010), especially if marsh is being claimed by increasing water levels while also being squeezed against natural or human barriers present in higher elevation areas.

## Conclusion

In this study, a modeling framework employs marsh migration predictions from SLAMM as parameterization into a hydrodynamic and wave model (ADCIRC + SWAN) capable of explicitly representing wave attenuation by vegetation under storm surge conditions. This approach was utilized to investigate how the storm protection benefits provided by salt marshes might evolve under distinct future climate change scenarios. Results show that potential resilience thresholds in the study area are strongly dependent on SLR scenarios (probability level and emission pathways) regardless of a specific point or period in time (i.e., timestamp) within the modeling experiment. Moreover, it is evident those temporal thresholds emerge where salt marsh extent sharply decreases and wave heights increase. Thus, indicating that utilizing a comprehensive range of SLR scenarios in marsh migration modeling can be strategic for better informing decision-making and guiding environmental policies in response to SLR-driven environmental changes.

## Supplementary Information

Supplementary Material 1.

## Data Availability

The field campaign-based vegetation data used for estimating average vegetation characteristics (height, stem density, and diameter) in the study are available at the Flood Hazards Research Lab CUAHSI HydroShare via 10.4211/hs.9034fa75324d46299984b681c54218e9, 10.4211/hs.f7350813a1de4025a3b8e4d05284dc57, 10.4211/hs.3ed0608d3bb84160a5714861632adcad, 10.4211/hs.61655dd48ac2435c976cf9bb8acefc92, and 10.4211/hs.b6d0ade225af4a3388e8bdb01f586c6c. The SLAMM marsh migration predictions are available at https://warrenpinnacle.com/prof/SLAMM/EESLR_MD/.
