# Supplementary material for: Assessing the potential long-term effects of sea-level rise on salt marsh’s coastal protective capacity under different climate pathway scenarios
Source: Environ Monit Assess. 2024 Aug 16;196(9):817. doi: 10.1007/s10661-024-12961-z (PMC11327207; doi:10.1007/s10661-024-12961-z)
Supplement: Supplementary file 1 — Supplementary Material 1. [file 10661_2024_12961_MOESM1_ESM.docx]

# Supplementary Material 1


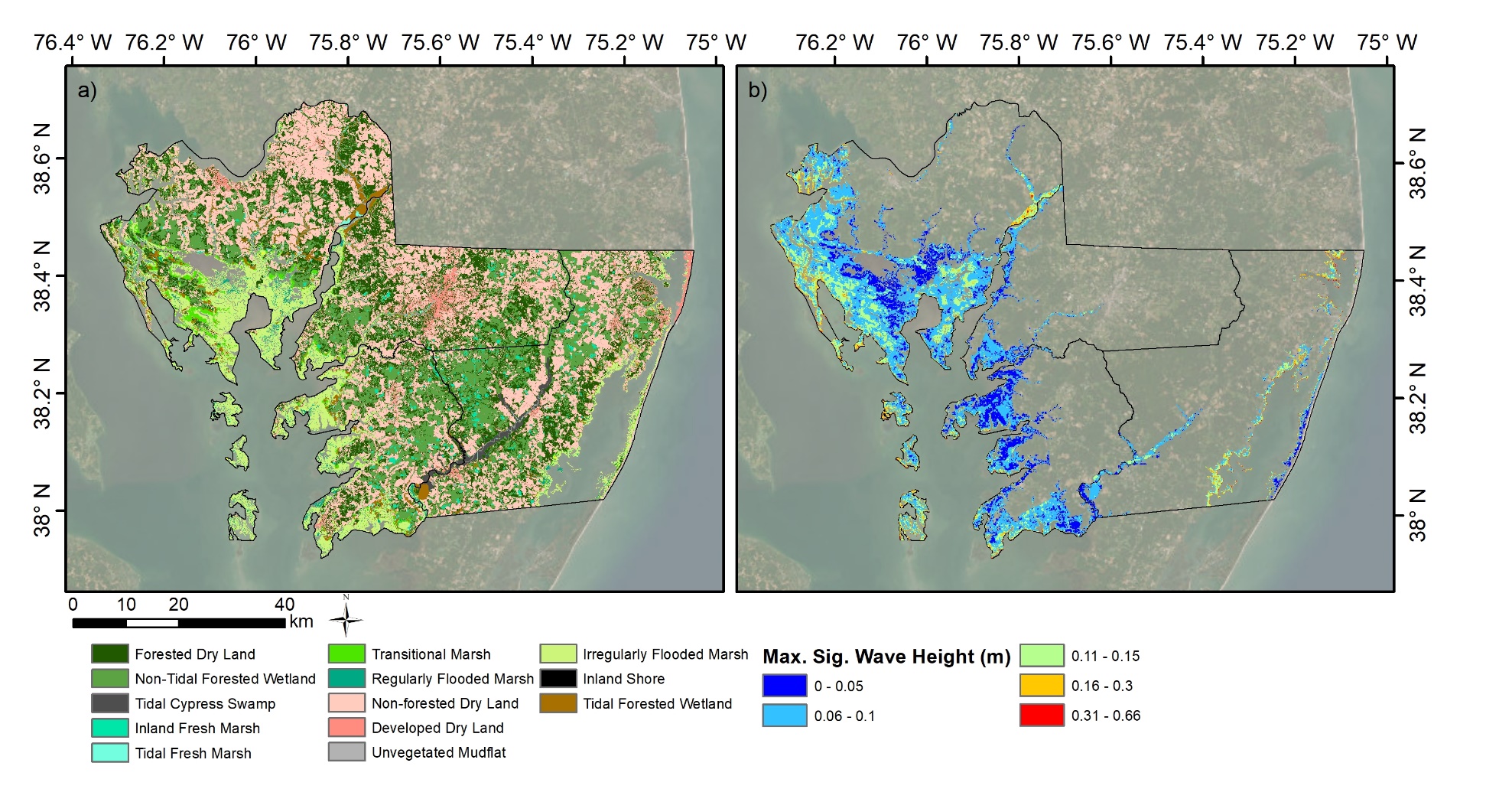


Figure 1: a) SLAMM classed under current conditions and, b) Overland maximum Significant Wave Height generated during Hurricane Isabel.

# Supplementary Material 2

Table 1: Summary of changes in marsh area and significant wave height (Hurricane Isabel) – 50% probability - RCP 4.5

| **County** | **Marsh area** | **Dry areas (excluding marsh)** | **Water and flooded areas** | **Scenario** | **Timestamp** | **Max. Hs** | **Avg. Hs** | **Med. Hs** | **90th Perc. Hs** |
| --- | --- | --- | --- | --- | --- | --- | --- | --- | --- |
| Wicomico | 50565200 | 906814900 | 8268600 | 50% probability (RCP 4.5) | 2010 | 0.51 | 0.07 | 0.06 | 0.10 |
| Dorchester | 336842700 | 1011366800 | 12665400 | 50% probability (RCP 4.5) | 2010 | 0.59 | 0.09 | 0.08 | 0.14 |
| Somerset | 198931300 | 594408300 | 5314800 | 50% probability (RCP 4.5) | 2010 | 0.55 | 0.07 | 0.06 | 0.13 |
| Worcester | 73943200 | 1116260400 | 12530300 | 50% probability (RCP 4.5) | 2010 | 0.66 | 0.11 | 0.10 | 0.20 |
| Wicomico | 66338100 | 888859300 | 10451300 | 50% probability (RCP 4.5) | 2050 | 0.42 | 0.04 | 0.03 | 0.09 |
| Dorchester | 457686500 | 868388700 | 34799700 | 50% probability (RCP 4.5) | 2050 | 0.71 | 0.06 | 0.04 | 0.13 |
| Somerset | 233155700 | 548460400 | 17038300 | 50% probability (RCP 4.5) | 2050 | 0.71 | 0.07 | 0.05 | 0.15 |
| Worcester | 81205700 | 1090317400 | 31210800 | 50% probability (RCP 4.5) | 2050 | 0.69 | 0.13 | 0.11 | 0.25 |
| Wicomico | 78250700 | 875198200 | 12199800 | 50% probability (RCP 4.5) | 2080 | 0.45 | 0.04 | 0.02 | 0.10 |
| Dorchester | 512254000 | 774970000 | 73650900 | 50% probability (RCP 4.5) | 2080 | 0.80 | 0.06 | 0.04 | 0.15 |
| Somerset | 260620300 | 510279200 | 27754900 | 50% probability (RCP 4.5) | 2080 | 0.79 | 0.07 | 0.05 | 0.16 |
| Worcester | 81157700 | 1068647500 | 52928700 | 50% probability (RCP 4.5) | 2080 | 0.77 | 0.17 | 0.14 | 0.31 |
| Wicomico | 83721100 | 868688400 | 13239200 | 50% probability (RCP 4.5) | 2100 | 0.48 | 0.04 | 0.02 | 0.10 |
| Dorchester | 522960900 | 732762800 | 105151200 | 50% probability (RCP 4.5) | 2100 | 0.86 | 0.07 | 0.04 | 0.16 |
| Somerset | 275152800 | 491674300 | 31827300 | 50% probability (RCP 4.5) | 2100 | 0.83 | 0.07 | 0.05 | 0.17 |
| Worcester | 76437900 | 1055488200 | 70807800 | 50% probability (RCP 4.5) | 2100 | 0.81 | 0.20 | 0.15 | 0.40 |

# Supplementary Material 3

Table 2: Summary of changes in marsh area and significant wave height (Hurricane Isabel) – 50% probability - RCP 8.5

| **County** | **Marsh area (m^2^)** | **Dry areas excluding marsh (m^2^)** | **Water and flooded areas (m^2^)** | **Scenario** | **Timestamp** | | **Max. Hs** | | **Avg. Hs** | | **Med. Hs** | | **90th Perc. Hs** | |
| --- | --- | --- | --- | --- | --- | --- | --- | --- | --- | --- | --- | --- | --- | --- |
| Wicomico | 50565200 | 906814900 | 8268600 | 50% probability (RCP 8.5) | 2010 | 0.51 | | 0.07 | | 0.06 | | 0.10 | |  |
| Dorchester | 336842700 | 1011366800 | 12665400 | 50% probability (RCP 8.5) | 2010 | 0.59 | | 0.09 | | 0.08 | | 0.14 | |  |
| Somerset | 198931300 | 594408300 | 5314800 | 50% probability (RCP 8.5) | 2010 | 0.55 | | 0.07 | | 0.06 | | 0.13 | |  |
| Worcester | 73943200 | 1116260400 | 12530300 | 50% probability (RCP 8.5) | 2010 | 0.66 | | 0.11 | | 0.10 | | 0.20 | |  |
| Wicomico | 66338100 | 888859300 | 10451300 | 50% probability (RCP 8.5) | 2050 | 0.42 | | 0.04 | | 0.03 | | 0.09 | |  |
| Dorchester | 457686500 | 868388700 | 34799700 | 50% probability (RCP 8.5) | 2050 | 0.71 | | 0.06 | | 0.04 | | 0.13 | |  |
| Somerset | 233155700 | 548460400 | 17038300 | 50% probability (RCP 8.5) | 2050 | 0.71 | | 0.07 | | 0.05 | | 0.15 | |  |
| Worcester | 81205700 | 1090317400 | 31210800 | 50% probability (RCP 8.5) | 2050 | 0.69 | | 0.13 | | 0.11 | | 0.25 | |  |
| Wicomico | 82552300 | 870168400 | 12928000 | 50% probability (RCP 8.5) | 2080 | 0.46 | | 0.05 | | 0.03 | | 0.10 | |  |
| Dorchester | 502531800 | 756900800 | 101442300 | 50% probability (RCP 8.5) | 2080 | 0.83 | | 0.07 | | 0.05 | | 0.16 | |  |
| Somerset | 272048700 | 496224400 | 30381300 | 50% probability (RCP 8.5) | 2080 | 0.82 | | 0.07 | | 0.05 | | 0.17 | |  |
| Worcester | 63616800 | 1064337100 | 74780000 | 50% probability (RCP 8.5) | 2080 | 0.80 | | 0.21 | | 0.17 | | 0.44 | |  |
| Wicomico | 88953100 | 861786900 | 14908700 | 50% probability (RCP 8.5) | 2100 | 0.50 | | 0.05 | | 0.03 | | 0.12 | |  |
| Dorchester | 416820100 | 769470500 | 174584300 | 50% probability (RCP 8.5) | 2100 | 0.93 | | 0.10 | | 0.08 | | 0.22 | |  |
| Somerset | 276120200 | 482414000 | 40120200 | 50% probability (RCP 8.5) | 2100 | 0.90 | | 0.09 | | 0.06 | | 0.21 | |  |
| Worcester | 55852700 | 1046457000 | 100424200 | 50% probability (RCP 8.5) | 2100 | 0.87 | | 0.26 | | 0.21 | | 0.53 | |  |

# Supplementary Material 4

Table 3: Summary of changes in marsh area and significant wave height (Hurricane Isabel) - Upper limit of the Likely Range - RCP 8.5

| **County** | **Marsh area (m^2^)** | **Dry areas excluding marsh (m^2^)** | **Water and flooded areas (m^2^)** | **Scenario** | **Timestamp** | | **Max. Hs** | | **Avg. Hs** | | **Med. Hs** | | **90th Perc. Hs** | |
| --- | --- | --- | --- | --- | --- | --- | --- | --- | --- | --- | --- | --- | --- | --- |
| Wicomico | 50565200 | 906814900 | 8268600 | Upper limit of the likely range (RCP 8.5) | 2010 | 0.51 | | 0.07 | | 0.06 | | 0.10 | |  |
| Dorchester | 336842700 | 1011366800 | 12665400 | Upper limit of the likely range (RCP 8.5) | 2010 | 0.59 | | 0.09 | | 0.08 | | 0.14 | |  |
| Somerset | 198931300 | 594408300 | 5314800 | Upper limit of the likely range (RCP 8.5) | 2010 | 0.55 | | 0.07 | | 0.06 | | 0.13 | |  |
| Worcester | 73943200 | 1116260400 | 12530300 | Upper limit of the likely range (RCP 8.5) | 2010 | 0.66 | | 0.11 | | 0.10 | | 0.20 | |  |
| Wicomico | 71739100 | 882580700 | 11328900 | Upper limit of the likely range (RCP 8.5) | 2050 | 0.43 | | 0.04 | | 0.03 | | 0.10 | |  |
| Dorchester | 485396400 | 828403300 | 47075200 | Upper limit of the likely range (RCP 8.5) | 2050 | 0.75 | | 0.06 | | 0.04 | | 0.14 | |  |
| Somerset | 244631400 | 531353600 | 22669400 | Upper limit of the likely range (RCP 8.5) | 2050 | 0.73 | | 0.07 | | 0.05 | | 0.16 | |  |
| Worcester | 76459400 | 1082954700 | 43319800 | Upper limit of the likely range (RCP 8.5) | 2050 | 0.73 | | 0.15 | | 0.13 | | 0.30 | |  |
| Wicomico | 80721900 | 869534900 | 15391900 | Upper limit of the likely range (RCP 8.5) | 2080 | 0.50 | | 0.06 | | 0.05 | | 0.13 | |  |
| Dorchester | 356043000 | 804636600 | 200195300 | Upper limit of the likely range (RCP 8.5) | 2080 | 0.92 | | 0.12 | | 0.10 | | 0.22 | |  |
| Somerset | 253655600 | 501699400 | 43299400 | Upper limit of the likely range (RCP 8.5) | 2080 | 0.89 | | 0.10 | | 0.07 | | 0.24 | |  |
| Worcester | 47481600 | 1055068200 | 100184100 | Upper limit of the likely range (RCP 8.5) | 2080 | 0.86 | | 0.27 | | 0.22 | | 0.54 | |  |
| Wicomico | 55578100 | 865908400 | 44162200 | Upper limit of the likely range (RCP 8.5) | 2100 | 0.57 | | 0.15 | | 0.14 | | 0.28 | |  |
| Dorchester | 139933500 | 731892900 | 489048500 | Upper limit of the likely range (RCP 8.5) | 2100 | 1.06 | | 0.25 | | 0.24 | | 0.41 | |  |
| Somerset | 135194500 | 508597600 | 154862300 | Upper limit of the likely range (RCP 8.5) | 2100 | 1.09 | | 0.25 | | 0.19 | | 0.54 | |  |
| Worcester | 46776200 | 1015975700 | 139982000 | Upper limit of the likely range (RCP 8.5) | 2100 | 1.01 | | 0.34 | | 0.28 | | 0.66 | |  |

# Supplementary Material 5

Table 4: Summary of changes in marsh area and significant wave height (Hurricane Isabel) - 1% probability- RCP 8.5

| **County** | **Marsh area (m^2^)** | **Dry areas excluding marsh (m^2^)** | **Water and flooded areas (m^2^)** | **Scenario** | **Timestamp** | | **Max. Hs** | | **Avg. Hs** | | **Med. Hs** | | **90th Perc. Hs** | |
| --- | --- | --- | --- | --- | --- | --- | --- | --- | --- | --- | --- | --- | --- | --- |
| Wicomico | 50565200 | 906814900 | 8268600 | 1% probability (RCP 8.5) | 2010 | 0.51 | | 0.07 | | 0.06 | | 0.10 | |  |
| Dorchester | 336842700 | 1011366800 | 12665400 | 1% probability (RCP 8.5) | 2010 | 0.59 | | 0.09 | | 0.08 | | 0.14 | |  |
| Somerset | 198931300 | 594408300 | 5314800 | 1% probability (RCP 8.5) | 2010 | 0.55 | | 0.07 | | 0.06 | | 0.13 | |  |
| Worcester | 73943200 | 1116260400 | 12530300 | 1% probability (RCP 8.5) | 2010 | 0.66 | | 0.11 | | 0.10 | | 0.20 | |  |
| Wicomico | 78978600 | 873774400 | 12895700 | 1% probability (RCP 8.5) | 2050 | 0.47 | | 0.05 | | 0.03 | | 0.11 | |  |
| Dorchester | 458333200 | 818154200 | 84387500 | 1% probability (RCP 8.5) | 2050 | 0.80 | | 0.08 | | 0.06 | | 0.16 | |  |
| Somerset | 259365800 | 509516200 | 29772400 | 1% probability (RCP 8.5) | 2050 | 0.79 | | 0.08 | | 0.05 | | 0.18 | |  |
| Worcester | 56050100 | 1077331200 | 69352600 | 1% probability (RCP 8.5) | 2050 | 0.78 | | 0.22 | | 0.17 | | 0.45 | |  |
| Wicomico | 44682800 | 858833700 | 62132200 | 1% probability (RCP 8.5) | 2080 | 0.60 | | 0.20 | | 0.19 | | 0.37 | |  |
| Dorchester | 107161200 | 709751500 | 543962200 | 1% probability (RCP 8.5) | 2080 | 1.09 | | 0.31 | | 0.30 | | 0.50 | |  |
| Somerset | 104397100 | 477738700 | 216518600 | 1% probability (RCP 8.5) | 2080 | 1.11 | | 0.32 | | 0.28 | | 0.61 | |  |
| Worcester | 46993500 | 1011999600 | 143740800 | 1% probability (RCP 8.5) | 2080 | 1.04 | | 0.37 | | 0.31 | | 0.71 | |  |
| Wicomico | 47654800 | 819017500 | 98976400 | 1% probability (RCP 8.5) | 2100 | 0.71 | | 0.27 | | 0.28 | | 0.50 | |  |
| Dorchester | 85185400 | 579007400 | 696682100 | 1% probability (RCP 8.5) | 2100 | 1.23 | | 0.47 | | 0.50 | | 0.70 | |  |
| Somerset | 93934000 | 382847100 | 321873300 | 1% probability (RCP 8.5) | 2100 | 1.34 | | 0.43 | | 0.43 | | 0.81 | |  |
| Worcester | 53711800 | 964197400 | 184824700 | 1% probability (RCP 8.5) | 2100 | 1.28 | | 0.49 | | 0.41 | | 0.93 | |  |

# Supplementary Material 6

Table 5: Conversions in salt marsh area by 2100

|  |  |  |  |  |  |  |  |  |  |
| --- | --- | --- | --- | --- | --- | --- | --- | --- | --- |
|  | **Marsh Loss (Converting To)** | | | | | | | |  |
|  | 50% Probability  (RCP 4.5) | Area (km^2^) | 50% Probability  (RCP 8.5) | Area (km^2^) | Upper Lim. Likely Range (RCP 8.5) | Area (km^2^) | 1% Probability (RCP 8.5) | Area (km^2^) |  |
|  | Unvegetated Mudflat | -9.4 | Unvegetated Mudflat | -78.5 | Unvegetated Mudflat | -120.1 | Unvegetated Mudflat | -0.3728 |  |
|  | Estuarine Open Water | **-92.4** | Estuarine Open Water | **-161.8** | Estuarine Open Water | **-502.0** | Estuarine Open Water | **-657.7** |  |
|  | Total Area (km^2^) | -101.8 |  | -240.3 |  | -622.1 |  | -658.0 |  |
|  |  |  |  |  |  |  |  |  |  |
|  | **New Marsh (Converted From)** | | | | | | | |  |
|  | 50% Probability  (RCP 4.5) | Area (km^2^) | 50% Probability  (RCP 8.5) | Area (km^2^) | Upper Lim. Likely Range (RCP 8.5) | Area (km^2^) | 1% Probability (RCP 8.5) | Area (km^2^) |  |
|  | Forested Dry Land | **127.6** | Forested Dry Land | 138.0 | Forested Dry Land | 120.9 | Forested Dry Land | 85.4 |  |
|  | Non-forested Dry Land | 124.6 | Non-forested Dry Land | **146.9** | Non-forested Dry Land | **149.7** | Non-forested Dry Land | **115.8** |  |
|  | Non-Tidal Forested Wetland | 69.2 | Non-Tidal Forested Wetland | 71.5 | Non-Tidal Forested Wetland | 61.9 | Non-Tidal Forested Wetland | 75.4 |  |
|  | Tidal Forested Wetland | 78.3 | Tidal Forested Wetland | 61.4 | Tidal Forested Wetland | 6.8 | Tidal Forested Wetland | 1.7 |  |
|  | Total Area (km^2^) | 399.8 |  | 417.7 |  | 339.3 |  | 278.3 |  |
